# Distribution of SERPINA1 gene mutations in patients with spontaneous pneumothorax: A cross-sectional study from a tertiary chest diseases clinic in Turkiye

**DOI:** 10.1097/MD.0000000000045043

**Published:** 2025-10-03

**Authors:** Savaş Gegin, Esra Arslan Aksu, Necmiye Gül Temel, Muhammet Ali Yilmaz, Deniz Çelik, Burcu Özdemir, Levent Özdemir

**Affiliations:** a Pulmonology Clinic, Samsun Training and Research Hospital, Samsun, Türkiye; b Pulmonology Department, Samsun University Faculty of Medicine, Samsun, Türkiye; c Department of Thoracic Surgery, Samsun University Faculty of Medicine, Samsun, Türkiye; d Department of Pulmonology, Alanya Alaaddin Keykubat Faculty of Medicine, Antalya, Türkiye.

**Keywords:** alpha-1 antitrypsin deficiency, genetic mutation, secondary pneumothorax, SERPINA1, spontaneous pneumothorax

## Abstract

Spontaneous pneumothorax (SP) is characterized by air accumulation between the visceral and parietal pleural layers without traumatic or iatrogenic causes. Although its etiology is not fully understood, risk factors include low body mass index, tall stature, smoking, and male sex. Alpha-1 antitrypsin deficiency (AATD), caused by *SERPINA1* mutations, may contribute to secondary SP (SSP) through mechanisms such as alveolar destruction and emphysema development. This study aimed to determine the frequency and distribution of *SERPINA1* gene mutations in individuals diagnosed with SP and to assess the distribution of these mutations according to pneumothorax type – primary SP (PSP) versus SSP. This cross-sectional descriptive study was conducted at the Pulmonology Clinic of Samsun Training and Research Hospital between January 1, 2022, and December 31, 2024. A total of 100 patients aged over 18 years who were diagnosed with SP and provided informed consent were included. Dried blood spot samples were collected for *SERPINA1*genotyping (AlphaKits® GE Healthcare Ltd, Cardiff, UK), performed at the Progenika Clinical Diagnostics Laboratory (Spain). Demographic characteristics, smoking status, pneumothorax type, and thoracic CT findings were analyzed. One hundred patients (86% male) with a mean age of 38.8 ± 17.2 years were included. Sixty-six percent were smokers, 23% were nonsmokers, and 11% were ex-smokers. Among them, 55% had SSP and 45% had PSP. The underlying cause of SSP was emphysema in 43 patients (78.1%) and bronchiectasis in 12 patients (21.9%). *SERPINA1* gene mutations associated with AATD were identified in 2 patients (2%), both in the SSP group. No mutations were detected in PSP cases. The genotype detected in both cases was PI*M/I, and both patients were smokers with emphysematous changes on thoracic CT. In conclusion, Screening strategies based solely on A1AT serum levels are insufficient as some carriers may have near-normal levels. However, routine genotyping of all SP patients is not supported by our data. Large prospective studies should clarify the role of targeted genetic testing in selected patient subgroups.

## 1. Introduction

Spontaneous pneumothorax (SP) is characterized by air accumulation between the visceral and parietal pleural layers without a traumatic or iatrogenic cause, and represents a common clinical condition in pulmonary practice. Clinically, it is divided into 2 main categories: primary SP (PSP) and secondary SP (SSP). In PSP, there is no underlying significant lung disease, whereas SSP is generally associated with chronic pulmonary diseases^[1]^

Although the etiology of PSP has not been fully elucidated, it is known to be associated with risk factors such as low body mass index, body habitus, tall stature, smoking, and male sex. Subpleural blebs and bullae are structural changes frequently encountered in this patient group and typically result in pneumothorax upon spontaneous rupture. SSP, on the other hand, most often develops in association with chronic obstructive pulmonary disease, bronchiectasis, emphysema, interstitial lung diseases, infections, fibrotic disorders, and, rarely, genetic syndromes.^[2]^

Alpha-1 antitrypsin (AAT) is the principal serine protease inhibitor produced by hepatocytes in the liver. It is responsible for inhibiting proteases generated during inflammatory or infectious states.^[3]^ Alpha-1 antitrypsin deficiency (AATD) is an autosomal co-dominant inherited disorder caused by mutations in the SERPINA1 gene, leading to reduced serum AAT levels. AATD may result in protease–antiprotease imbalance, alveolar damage, and consequently panacinar emphysema, chronic obstructive pulmonary disease, and bronchiectasis.^[4]^

While many mechanisms play a role in the etiology of SP, there is no clear consensus in the literature regarding its relationship with AATD. The alveolar wall destruction, emphysema, and bulla formation caused by AATD in the lung may predispose to pneumothorax. However, studies evaluating the association between these 2 clinical conditions are limited. Most existing studies have examined only serum AAT levels without genetic analysis.^[5–7]^ Since AAT is an acute-phase reactant, its level may increase during inflammation or infection, potentially confounding the diagnosis. Therefore, genetic mutation analyses may provide more reliable and enduring diagnostic results.

This study was designed to determine the frequency and distribution of mutations in the SERPINA1 gene among individuals diagnosed with SP. Additionally, by performing a comparative analysis of AATD genotypes according to pneumothorax type (primary vs secondary), it aims to contribute to a better understanding of the potential clinical impact of alpha-1 antitrypsin deficiency.

## 2. Materials and methods

The study was conducted as a cross-sectional descriptive study by evaluating the data of 100 patients with SP at the Pulmonology Clinic of Samsun Training and Research Hospital between January 1, 2022 and December 31, 2024. Patients over 18 years of age with pneumothorax who provided informed consent and agreed to participate were included.

For AAT genotyping, dried blood spot samples obtained from fingertip capillary blood were used. Genotyping (AlphaKits® GE Healthcare Ltd, Cardiff, CF14 7YT, UK) was performed at the Progenika Clinical Diagnostics Laboratory in Spain. The alleles analyzed in patients included PII, PIM Procida, PIM Malton, PIS Iiyama, PIQ0 Granite Falls, PIQ0 West, PIQ0 Bellingham, PIF, PIP Lowell, PIS, PIZ, PIQ0 Mattawa, PIQ0 Clayton, and PIM Heerlen.

During screening, patients’ demographic characteristics (age, sex), smoking status (smoker, ex-smoker, nonsmoker), pneumothorax type (primary, secondary), side of pneumothorax (right, left), and number of pneumothorax episodes were recorded. All patients underwent thoracic computed tomography to evaluate parenchymal lung disease. Pneumothorax types were classified as follows:

PSP: pneumothorax in patients without underlying parenchymal lung disease^[1]^

SSP: pneumothorax in patients with underlying parenchymal lung disease^[1]^

In patients found to have AAT genotypic deficiency, serum AAT levels were also measured and evaluated.

This study was conducted in accordance with the principles of the Declaration of Helsinki. Ethical approval was obtained from the Samsun University Clinical Research Ethics Committee (Approval Number: 2025/2/1, Date: 24.01.2025). Written informed consent was obtained from all participants prior to inclusion in the study.

## 3. Statistical analysis

All analyses were performed using SPSS 22 for Windows (SPSS Inc., Chicago). Categorical variables were summarized as frequency and percentage (%), and continuous variables as mean, median, and standard deviation.

## 4. Results

Between January 2022 and December 2024, a total of 100 patients with SP (mean age 38.8 ± 17.2 years; male 37.9 ± 17.1 years, female 44.1 ± 17.4 years) were included in the study. Fourteen patients (14%) were female and 86 (86%) were male. According to smoking status, 66 patients (66%) were smokers, 23 (23%) were nonsmokers, and 11 (11%) were ex-smokers.

Regarding pneumothorax type, 55 patients (55%) had SSP and 45 (45%) had PSP. Among SSP patients, the underlying cause was emphysema in the majority (n = 43; 78.1%), while bronchiectasis was identified in the remaining 12 patients (21.9%) (Table 1).

**Table 1:** Patient demographic and clinical characteristics.

Gender	Female n (%) 14 (14)	Male n (%) 86 (86)	Total n 100
Age	44.1 ± 17.4	37.9 ± 17.1	38.8±/17.2
Smoking status			
Smoker	6 (6)	60 (60)	66 (66)
Nonsmoker	7 (7)	16 (16)	23 (23)
Ex-smoker	1 (1)	10 (10)	11 (11)
Pneumothorax type			
Primary spontaneous pneumothorax	5 (5)	40 (40)	45 (45)
Secondary spontaneous pneumothorax	9 (9)	46 (46)	55 (55)
Emphysema	5	38	43
Bronchiectasis	4	8	12

**Figure 1.**
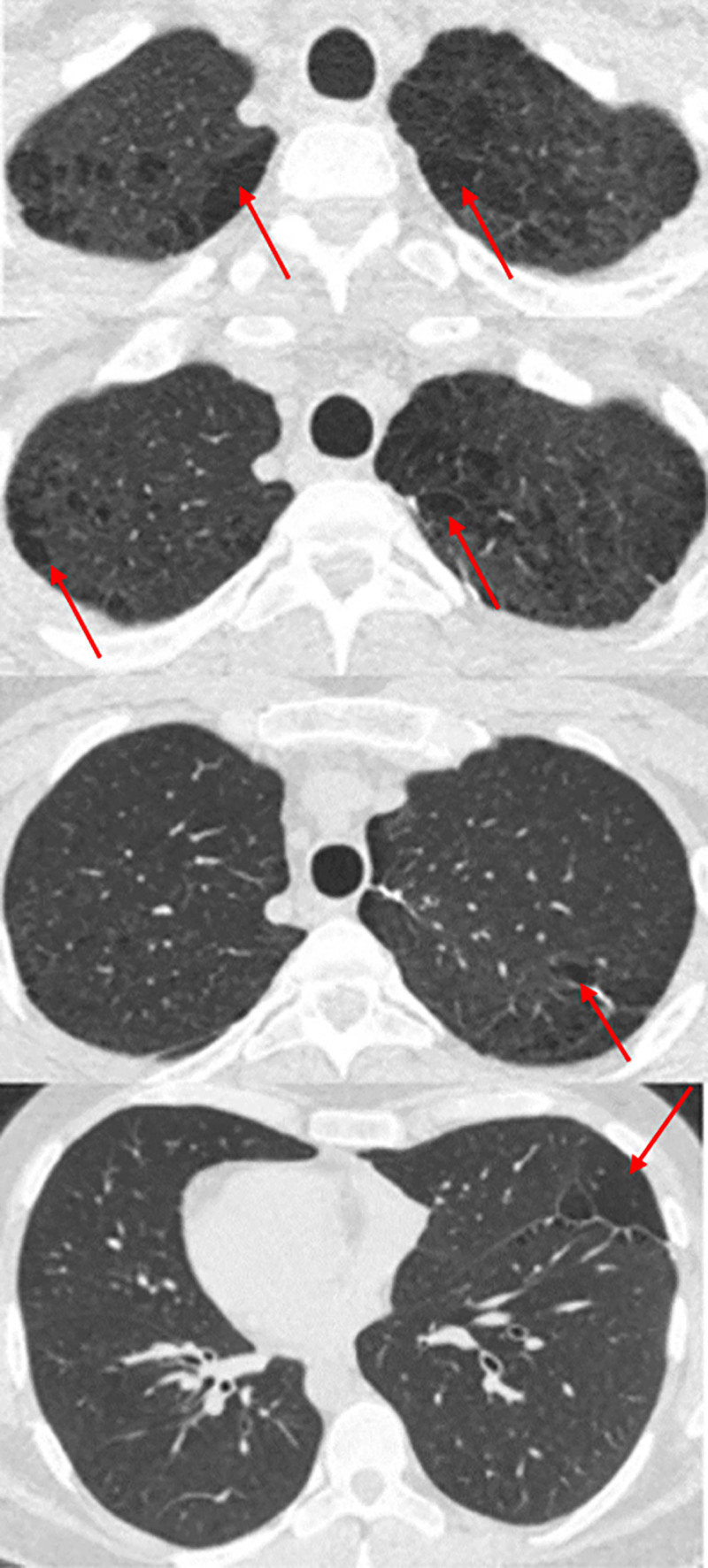
Thoracic computed tomography of the patient with secondary SP. SP = spontaneous pneumothorax.

According to the AAT genotyping results, no mutation was detected in 98 patients (98%), whereas 2 patients (2%) were found to have genetic mutations associated with alpha-1 antitrypsin deficiency (AATD). Both AATD cases were in the SSP group, and no AATD was detected in the PSP. The SERPINA1 genotype of both patients with mutations was determined as PI*M/I. The serum AAT levels of the 2 patients with detected mutations were 1.41 g/L and 1.27 g/L, respectively. The characteristics of patients with SSP are shown in Table 2 (see Fig. 1, case 1).

**Table 2:** Clinical characteristics of patients diagnosed with alpha-1 antitrypsin deficiency.

	Genotype	Gender/ Age	Cigarete	Pneumothorax right/left	Pneumothorax times	AAT level N:0, 9–2 g/L
Case 1	PI*M/I	M/28	Smoker	R/L	2/2	1.41
Case 2	PI*M/I	M/38	Smoker	R	1	1.27

## 5. Discussion

This cross-sectional descriptive study was designed to investigate the presence of mutations in the SERPINA1 gene in patients diagnosed with SP. In our study, no mutations were detected in any patients with PSP, whereas SERPINA1 gene mutations were identified in 2 patients with SSP. It is notable that both patients were smokers and exhibited parenchymal lung disease findings on thoracic CT suggestive of underlying emphysema.

Current guidelines do not recommend routine screening for alpha-1 antitrypsin deficiency (AATD) in cases of SP. However, the prevalence and clinical characteristics of AATD in patients with SP remain incompletely defined. Most studies in the literature focus solely on serum AAT levels, and genotyping is applied only to cases with low serum levels.^[5–7]^ In this context, our study is one of the few in which genetic analysis results were applied to and directly reported for the entire cohort.

In a prospective cross-sectional study by Menga et al, 58 patients with PSP were evaluated. Genotyping was performed on 15 patients (25.9%) with serum AAT levels ≤ 120 mg/dL, and genetic variants associated with AAT deficiency were detected in 7 of these patients (12%). The reported genotype distribution was PiZZ (n = 1), heterozygous PiZ (n = 3), and heterozygous Pi*S (n = 3).^[5]^ In the study by Serapinas et al, serum AAT levels and SERPINA1 genotypes of 39 patients with SP were compared with those of 100 healthy individuals. AAT levels were found below the reference limits in 3 patients (7%) in the SP group and in 1 individual (1%) in the control group. However, no pathogenic genetic mutations associated with alpha-1 antitrypsin deficiency (AATD) were detected in any of these low–serum–level individuals.^[6]^ In the study conducted by Dias et al, 103 patients with PSP were evaluated, and 1 patient (1.9%) was found to have the Pi*SZ genotype. Based on these findings, they suggested that genetic mutations may play a non-negligible role in the etiology of PSP and emphasized that genetic analysis in certain subgroups could be clinically beneficial.^[7]^ In the study conducted by Pawlowicz et al on 56 patients with SP and 20 healthy individuals, the relationship between alpha-1 antitrypsin deficiency (AATD) and SP was investigated. No significant difference in serum AAT levels was observed between the patient and control groups. They concluded that AATD does not have a meaningful place in the routine etiological workup of idiopathic SP cases and proposed that genotyping may be useful only in cases with low serum AAT levels.^[8]^ In the study by Chen et al, which compared 32 patients with SP to healthy individuals, it was found that during SP attacks, patients’ serum alpha-1 antitrypsin (AAT) levels were significantly higher than those of healthy controls. Therefore, they emphasized that diagnosing AAT deficiency (AATD) based solely on serum AAT levels is unreliable and that genetic testing is essential for accurate detection of AATD in SP patients.^[9]^ A common limitation of these studies is that genotyping was performed only in individuals with low serum AAT levels or was not performed at all. Moreover, most studies focus on PSP cases, and data on SSP are quite limited. Although the literature generally reports an AATD prevalence of 1% to 2%, researchers such as Dias and Menga have highlighted the value of genetic analysis in patients who are smokers, have emphysema findings, and have a history of recurrent SP episodes. In our study, no mutations were detected in primary SP cases; however, In the 2 patients diagnosed with SSP, the PI*M/I genotype may not be a strong risk factor on its own, but it could become clinically significant when combined with smoking and emphysema. This underscores the importance of conducting genetic screening based on selected clinical features rather than relying solely on serum AAT levels.

This study has several limitations. First, it is a single-center, retrospective design, and the small sample size limits the generalizability of the findings. The commercial kit used in our study screens only for known common and some rare SERPINA1 variants, and therefore may not have detected all novel mutations. In addition, since no ambiguous genotypic findings were observed in our results, no additional confirmatory testing was performed. Although genetic analysis was performed on all participants, there is no comparative control group. Nevertheless, the strengths of this study include the use of direct genetic screening instead of serum AAT levels and the evaluation of parenchymal lung diseases by thoracic CT.

In conclusion, Screening strategies based solely on A1AT serum levels are insufficient as some carriers may have near-normal levels. However, routine genotyping of all SP patients is not supported by our data. Large prospective studies should clarify the role of targeted genetic testing in selected patient subgroups.

## Author contributions

**Conceptualization:** Savaş Gegin, Necmiye Gül Temel, Muhammet Ali Yilmaz, Deniz Çelik, Burcu Özdemir, Levent Özdemir.

**Data curation:** Savaş Gegin, Esra Arslan Aksu, Deniz Çelik, Burcu Özdemir, Levent Özdemir.

**Formal analysis:** Savaş Gegin, Esra Arslan Aksu, Necmiye Gül Temel, Muhammet Ali Yilmaz.

**Funding acquisition:** Savaş Gegin, Muhammet Ali Yilmaz, Deniz Çelik.

**Investigation:** Savaş Gegin, Esra Arslan Aksu, Burcu Özdemir.

**Methodology:** Savaş Gegin, Esra Arslan Aksu, Muhammet Ali Yilmaz, Deniz Çelik.

**Project administration:** Savaş Gegin.

**Resources:** Savaş Gegin, Esra Arslan Aksu, Muhammet Ali Yilmaz, Burcu Özdemir, Levent Özdemir.

**Software:** Savaş Gegin, Necmiye Gül Temel, Burcu Özdemir, Levent Özdemir.

**Supervision:** Savaş Gegin, Esra Arslan Aksu, Necmiye Gül Temel, Muhammet Ali Yilmaz, Deniz Çelik, Burcu Özdemir.

**Validation:** Savaş Gegin, Necmiye Gül Temel, Muhammet Ali Yilmaz, Deniz Çelik, Levent Özdemir.

**Visualization:** Savaş Gegin.

**Writing – original draft:** Savaş Gegin.

**Writing – review & editing:** Savaş Gegin, Esra Arslan Aksu, Necmiye Gül Temel, Muhammet Ali Yilmaz, Deniz Çelik, Burcu Özdemir, Levent Özdemir.
